# Nanoscale, antigen encounter-dependent, IL-12 delivery by CAR T cells plus PD-L1 blockade for cancer treatment

**DOI:** 10.1186/s12967-023-04014-9

**Published:** 2023-02-28

**Authors:** Zhifen Yang, Violena Pietrobon, Maggie Bobbin, Ofir Stefanson, Jin Yang, Angshumala Goswami, Bennett Alphson, Hana Choi, Khristina Magallanes, Qi Cai, David Barrett, Bing Wang, Lei S. Qi, Francesco M. Marincola

**Affiliations:** 1 Refuge Biotechnologies Inc., Menlo Park, CA 94025 USA; 2 Kite Pharma Inc., Santa Monica, CA 90404 USA; 3 Department of Bioengineering, Department of Chemical and Systems Biology, Stanford University, ChEM-H, Stanford, CA 94305 USA

**Keywords:** CRISPRa, Synthetic biology, Non-gene editing, Armored CAR-T cells, Tumor microenvironment

## Abstract

**Background:**

Chimeric antigen receptor (CAR)-T cell therapies for the treatment of hematological malignancies experienced tremendous progress in the last decade. However, essential limitations need to be addressed to further improve efficacy and reduce toxicity to assure CAR-T cell persistence, trafficking to the tumor site, resistance to an hostile tumor microenvironment (TME), and containment of toxicity restricting production of powerful but potentially toxic bioproducts to the TME; the last could be achieved through contextual release upon tumor antigen encounter of factors capable of converting an immune suppressive TME into one conducive to immune rejection.

**Methods:**

We created an HER2-targeting CAR-T (RB-312) using a clustered regularly interspaced short palindromic repeats (CRISPR) activation (CRISPRa) system, which induces the expression of the IL-12 heterodimer via conditional transcription of its two endogenous subunits p35 and p40. This circuit includes two lentiviral constructs. The first one (HER2-TEV) expresses an anti-human epidermal growth factor receptor 2 (HER2) CAR single chain variable fragment (scFv), with CD28 and CD3z co-stimulatory domains linked to the *tobacco etch virus* (TEV) protease and two single guide RNAs (sgRNA) targeting the interleukin (IL)-12A and IL12B transcription start site (TSS), respectively. The second construct (LdCV) encodes linker for activation of T cells (LAT) fused to nuclease-deactivated Streptococcus Pyogenes Cas9 (dCas9)-VP64-p65-Rta (VPR) via a TEV-cleavable sequence (TCS). Activation of the CAR brings HER2-TEV in close proximity to LdCV releasing dCas9 for nuclear localization. This conditional circuit leads to conditional and reversible induction of the IL-12/p70 heterodimer. RB-312 was compared in vitro to controls (cRB-312), lacking the IL-12 sgRNAs and conventional HER2 CAR (convCAR).

**Results:**

The inducible CRISPRa system activated endogenous IL-12 expression resulting in enhanced secondary interferon (FN)-γ production, cytotoxicity, and CAR-T proliferation in vitro, prolonged in vivo persistence and greater suppression of HER2^+^ FaDu oropharyngeal cancer cell growth compared to the conventional CAR-T cell product. No systemic IL-12 was detected in the peripheral circulation. Moreover, the combination with programmed death ligand (PD-L1) blockade demonstrated robust synergistic effects.

**Conclusions:**

RB-312, the first clinically relevant product incorporating a CRISPRa system with non-gene editing and reversible upregulation of endogenous gene expression that promotes CAR-T cells persistence and effectiveness against HER2-expressing tumors. The autocrine effects of reversible, nanoscale IL-12 production limits the risk of off-tumor leakage and systemic toxicity.

**Supplementary Information:**

The online version contains supplementary material available at 10.1186/s12967-023-04014-9.

## Introduction

Chimeric antigen receptor (CAR)-T cell therapy experienced tremendous progress in the last decade as an effective strategy targeting B cell malignancies [1] and multiple myeloma [2]. However, major hindrances still need to be faced to improve CAR-T cell efficacy, especially against solid tumors. Clinical benefits are in part limited by a tumor microenvironment (TME) presenting physical, functional and dynamic barriers that hamper CAR-T cell infiltration and by immune suppressive mechanisms that dampen their function [3–5]. Such hurdles are surmountable by enhancements that can turn an immune suppressive into an immune active TME; among them interleukin (IL)-12, a potent inducer of interferon (FN)-γ [6, 7], can play a prominent role if systemic toxicity can be mitigated [8]. Therefore, nanoscale delivery of IL-12 restricted to the TME presents a promising solution.

Modern gene editing technology provides formidable tools to regulate CAR-T transcriptional programs via knock-out or knock-in of transgenes, to reduce undesirable and enhance desirable properties [4]. However, genome-editing nucleases such as TALENs, zinc finger nucleases (ZFN) and clustered regularly interspaced short palindromic repeats (CRISPR)-associated systems (Cas) present their own limitations including permanent genomic alterations such as gross chromosomal rearrangements, a natural byproduct of simultaneous double strand breaks. The problem is compounded by multiple gene editing required when several functions need to be modulated simultaneously [9]. Therefore, a non-gene editing inducible system leading to transient and context-dependent modulation of cellular functions is preferable.

We previously described a non-editing expression regulation tool based on the nuclease de-activated CRISPR-associated (dCas9) protein, which does not cause structural genetic alterations but acts as a platform for RNA-guided DNA targeting [10–12]. We also reported that dCas9 can be fused the Kruppel associated box domain (KRAB) transcriptional repressor to target specific gene regulatory regions in the presence of a relevant single guide RNA (sgRNA). Once KRAB approaches to the promoter region, it downregulates transiently the expression of the target gene. We designed such construct to be inducible upon T cell activation to successfully downregulate PD-1 gene expression, a system referred to as CRISPR interference (CRISPRi). The engineered CAR-T cell product demonstrated enhanced cellular persistence and tumor eradication [13].

IL-12, a heterodimeric protein consisting of the p35 and p40 subunits, is a pleiotropic proinflammatory cytokine with potent tumor-suppressive activity that represents a promising candidate for combinatorial immunotherapy [6–8, 14]. IL-12 is involved in the differentiation of naïve T cells into Th1 cells, and activation of T cells and NK cells, leading to their proliferation, increased production of IFN-γ and cytotoxic potential. Combination of CAR-T cells with IL-12 enhances anti-tumor response in various pre-clinical models [15–21]. However, systemic delivery of IL-12 is poorly tolerated and can lead to fatalities during clinical trials [22]. Administration of armored T cells genetically engineered to expresses IL-12 is also associated with severe toxicity [23]. Intra-tumoral IL-12 delivery in combination with CAR-T cell therapy in glioblastoma appeared to be less toxic [24]. A human study administering tumor infiltrating lymphocytes (TIL) genetically engineered to secrete IL-12 selectively at the tumor site demonstrated limited success due to overwhelming toxicities despite the low doses of TIL administered, about 100-fold lower than conventional TIL therapy in the absence of systemic administration of human recombinant IL-2 [23]. Conditionality was hypothesized by the transduction of a single chain IL-12 transgene under an NFAT-responsive promoter presuming NFAT expression only in response to antigen-encounter by the endogenous T cell receptor in the TME. However, toxicities were related to a non-controllable secretion of IL-2, which initiated a self-perpetuating autocrine/paracrine activation resulting in lethal systemic levels of IL-12 and IFN-γ. High levels of non-specific IL-12 secretion in vivo may have also been due to the random introduction of the *IL12* gene into T cells with other reactivities, such as those against viral antigens. Such reactivities may be capable of stimulating the cells in vivo, resulting in the regulation of the expression of NFAT. Therefore, localized, truly conditional, and finely tuned secretion of IL-12 in the TME is critical.

In this paper, we describe a novel HER2 CAR-T product, RB-312, using a CRISPR activation (CRISPRa) system, which express dCas9 fused to the transcriptional activator domains VP64-p65-Rta (VPR) and two sgRNAs targeting to two endogenous genes, *IL12A* and *IL12B*, encoding p35 and p40 respectively. Activation of RB-312 upon tumor encounter releases dCas9-VPR from cytoplasmic tethering, leading to transient transcription and expression of p35 and p40, that dimerize to form biologically active IL-12/p70. We thus created a synthetic CAR-T cell product, RB-312, that delivers tumor antigen-dependent, locally induced IL-12 with tumor growth suppressive effects and no potential risk for systemic effects. Our results also demonstrate the rationale for a combination therapy of PD-1/PD-L1 blockade with RB-312.

## Results

### Conditional release of IL-12/p70 by RB-312

**Fig. 1:**
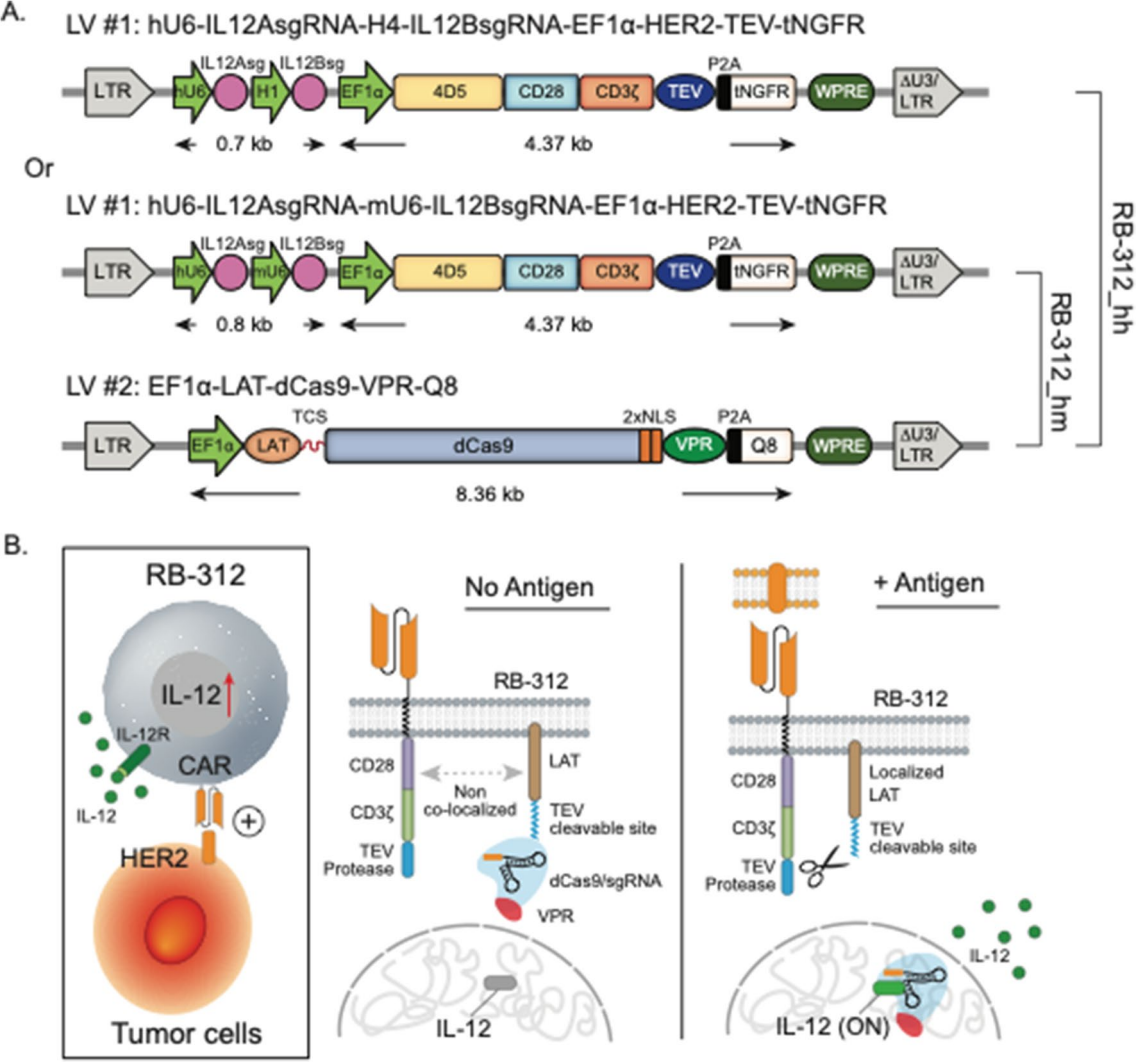
**A** Lentiviral components of RB‐312. RB-312 includes two lentiviral (LV) constructs. LV#1 (HER2‐TEV) encodes an anti‐HER2 (4D5 clone) scFv combined to the CD28 and CD3ζ co‐stimulatory domains, the TEV protease and IL12Asg and IL12Bsg targeting the TSS of the endogenous IL12A (encoding p35 subunit) and IL12B (encoding p40 subunit) genes, which are driven by human U6 (hU6) and H1 promoters (named “RB‐312_hh”) or hU6 and mouse U6 (mU6) promoters (named “RB‐312_hm”). LV#2 (LdCV) encodes LAT, fused to dCas9‐VPR via a TEV‐cleavable sequence (TCS). Bicistronic expression of tNGFR and Q8 was used for detection of HER2-TEV and LdCV, respectively. **B** Mechanism of activation of HER2‐TEV/LdCV CRISPRa platform—Activation of HER2 CAR brings TEV in proximity of LdCV releasing dCas9‐VPR for nuclear translocation to the IL12A and ***IL12B*** TSS and conditionally and reversibly upregulated IL-12/p70 heterodimer expression. The figure represents the CRISPRa logic at steady‐state levels of LdCV expression. However, LdCV expression varies according to the physio‐metabolic status of individual cells resulting in variable degree of transcriptional activity and consequently different substrate availability for TEV cleavage

The RB-312 CRISPR activation circuit includes two lentiviral constructs (Fig. 1A). The first construct (LV#1, HER2-TEV) expresses an anti-human HER2 CAR single chain variable fragment (scFv), with CD28 and CD3z co-stimulatory domains linked to the *tobacco etch virus* (TEV) protease and two single guide RNAs (sgRNA) targeting the IL12A and IL12B transcription start site (TSS), respectively. IL-12 is a heterodimer composed of a p35 (*IL12A* gene) and p40 (*IL12B* gene) subunits, therefore two sgRNAs are included. The second construct (LdCV) encodes linker for activation of T cells (LAT) fused to nuclease-deactivated spCas9 (dCas9)-VP64-p65-Rta (VPR) via a TEV-cleavable sequence (TCS). The CAR-T cell product containing the sgRNAs for IL-12 is named RB-312 while the corresponding identical construct lacking the sgRNAs is called control RB-312 (cRB-312).

Upon antigen encounter, the phosphorylated immune tyrosinase-based activation motif (ITAM) domains of the zeta chain activates ZAP70 that in turn phosphorylates LAT acting as a docking site that carries LdCV close to the TEV protease. This in turns leads to the cleavage of the TSC at the linker releasing of dCas9-VPR from cytoplasmic tethering (Fig. 1B). The latter then enters the nucleus, binds to the sgRNAs to dock at the respective endogenous IL-12 gene loci leading to the induction of gene expression.

**Fig. 2:**
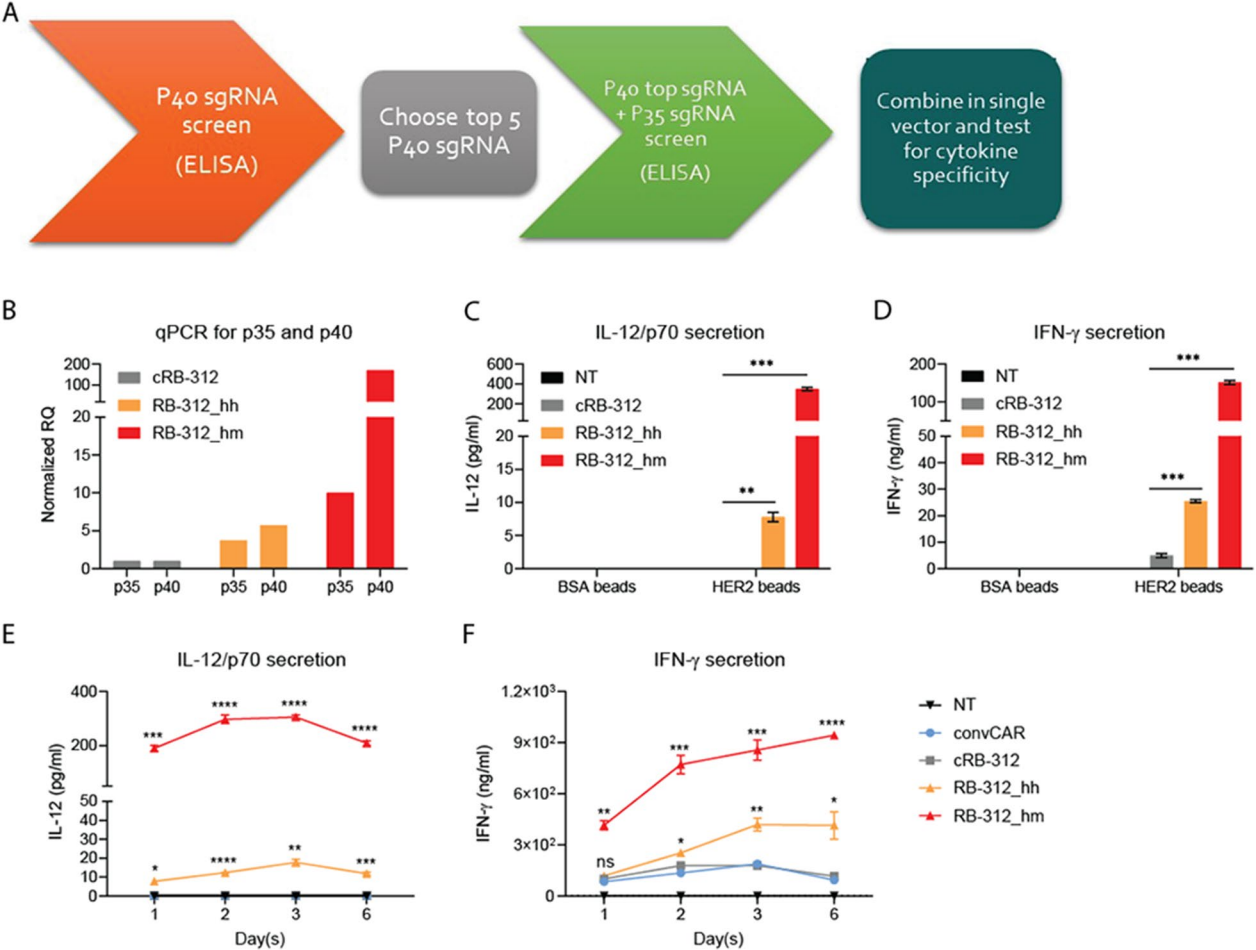
**A** Schematic of sgRNA screening for the IL-12 subunits p40 and p35 performed in this study. **B**, **C** Inducible upregulation of IL-12—Non‐transduced (NT), cRB‐312, RB‐312_hh and RB-312_hm cells were stimulated at a 1:1 bead to cell ratio with beads coated with bovine serum albumin (BSA beads) and HER2 ectodomain (HER2 beads). qPCR and IL-12/p70 ELISA were performed to monitor IL-12 mRNA subunits and heterodimer upregulation after 3-day incubation with beads. **D** IFN‐γ secretion tested after 3-day incubation with beads. **E**, **F** NT, cRB‐312, RB‐312_hh, RB-312_hm and conventional HER2 CAR (convCAR) were co-cultured with FaDu cells at 3:1 effector to target (E:T) ratio and tested for kinetics of IL-12/p70 expression and IFN‐γ secretion. *p* values are ranked by the asterisks in all panels (*p < 0.05, **p < 0.01, ***p < 0.001, ****p < 0.0001)

Arrayed guide RNA screening for the IL-12 subunits was performed as described in the material and methods; the top two sgRNAs for the p35 and p40 subunits were selected (Fig. 2A). Two configurations of the plasmid harboring the IL-12 sgRNAs were created. In the first one, sgRNAs for IL12A (encoding p35 subunit) and IL12B (encoding p40 subunit) are driven by two different human RNA polymerase III (Pol III) promoters human U6 (hU6) and H1, respectively (Fig. 1A). Although both promoters are often utilized in tandem to drive small nuclear RNA (snRNA) expression, U6 is supposed to be more potent than H1 [25]. To create equally strong expression for both IL12 sgRNAs, we thus designed a second version in which p35 sgRNA is under the control of hU6 while the p40 subunit is under the control of the murine U6 promoter (mU6). We called these two versions of RB-312: RB-312_hh and RB-312_hm, (Fig. 1A).

To induce IL-12 secretion, we stimulated for 3 days RB-312 and cRB-312 with HER2-coated beads on a 1:1 bead-to-cell ratio in T cell basal media (TBM) as previously described [26]. As control we used bovine serum albumin (BSA)-coated beads. Expression of both IL12A and IL12B was documented by real-time quantitative PCR (qPCR) (Fig. 2B). Importantly, RB-312_hm induced ~ 30-fold higher expression of IL12B and 2 ~ threefold higher expression of IL12A than RB-312_hh. This was confirmed by testing IL-12/p70 protein secretion in supernatants collected 3-days following HER2 beads stimulation (Fig. 2C). As expected, RB-312_hm displayed > 40-fold higher level of IL-12/p70 secretion compared to RB-312_hh, while the latter consistently displayed nanoscale range of IL-12/p70 secretion (5 ~ 20 pg/ml).

The pro-inflammatory cytokine IL-12 is a potent IFN-γ inducer [6, 7]. To confirm the biological activity of the secreted IL-12 in our system, we therefore examined IFN-γ secretion in the same specimens. Indeed, IFN-γ (ng/ml) was strongly induced in both RB-312_hh and RB-312_hm compared to control cRB-312. Moreover, RB-312_hm induced ~ sixfold higher IFN-γ than RB-312_hh (Fig. 2D).

Next, we stimulated RB-312 with the HER2^+^ FaDu oropharyngeal cancer cells. Following three days of co-culture, both IL-12 and IFN-γ were significantly induced in RB-312_hh and RB-312_hm (Fig. 2E and 2F). Again, RB-312_hm demonstrated dramatically higher induction of IL-12 (15 ~ 20-fold) and IFN-γ (2 ~ fourfold) than RB-312_hh. We thus created a conditionally, antigen-dependent IL-12 delivery system with high or low IL-12 secretion. Considering that high IL-12 delivery bears higher potential for severe systemic toxicity, in subsequent experiments we focused on testing the efficacy of the low-dose delivery version of RB-312_hh for in vitro and in vivo functions and for simplicity refer to it hereafter as RB-312.

### Nanoscale-delivery of IL-12/p70 by RB-312 enhances cytotoxicity and CAR-T cell expansion in vitro and in vivo

**Fig. 3:**
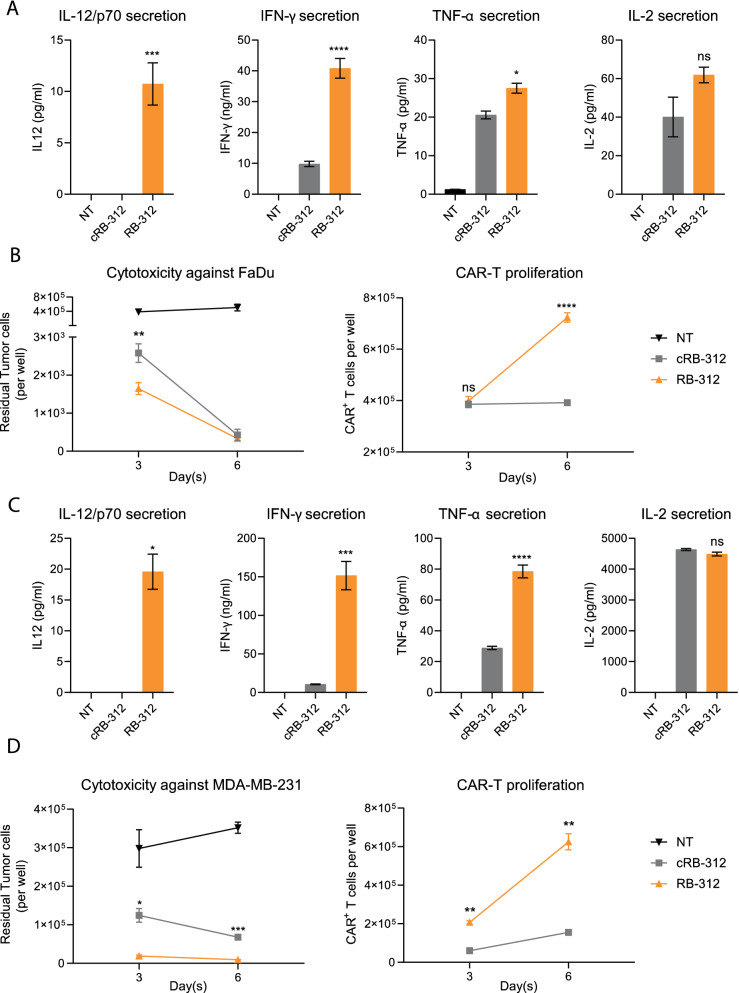
Modulation of cellular function by RB‐312. IL-12/p70, IFN‐γ, TNF‐α and IL‐2 secretion after 48-h stimulation with FaDu cells (**A**) or MDA-MB-231 cells (**C**) at 1:1 E:T ratio and cytotoxic activity and CAR‐T cell expansion were monitored at day 3 and 6 (**B**, **D**)

RB-312 and respective controls were tested in vitro against HER2^+^ FaDu oropharyngeal cancer cells or triple-negative breast cancer cells MDA-MB-231, both of which were engineered to constitutively express programmed cell death ligand-1 (PD-L1) to stabilize the cancer cell-dependent dynamics of PD-1/PD-L1 interactions (named FaDu and MDA-MB-231 hereafter). RB-312 induced nanoscale-dose IL-12/p70 secretion upon exposure to FaDu (Fig. 3A) or MDA-MB-231 cells (Fig. 3C) and significantly increased IFN-γ and tumor necrosis factor (TNF)-α secretion compared to cRB-312. CAR T cell expansion and cytotoxicity was also significantly enhanced in RB-312 (Fig. 3B and D). Higher CAR-T expansion was especially apparent 6 days following stimulation, although IL-2 production was not significantly different between RB-312 and the control cRB-312.

To recapitulate the tumor suppressive function of IL-12 in the FaDu tumor model, we also created a FaDu cell line overexpressing both PD-L1 and IL-12 (named FaDu_IL12) as a model system producing massive high IL-12 secretion (Additional file 1: Fig. S1A). Significantly higher cytotoxicity, better CAR-T expansion, enhanced IFN-γ and TNF-α secretion were observed when conventional HER2 CAR-T cells were co-culture with FaDu_IL12 compared to with FaDu tumor cells (Additional file 1: Fig. S1B–E).

**Fig. 4:**
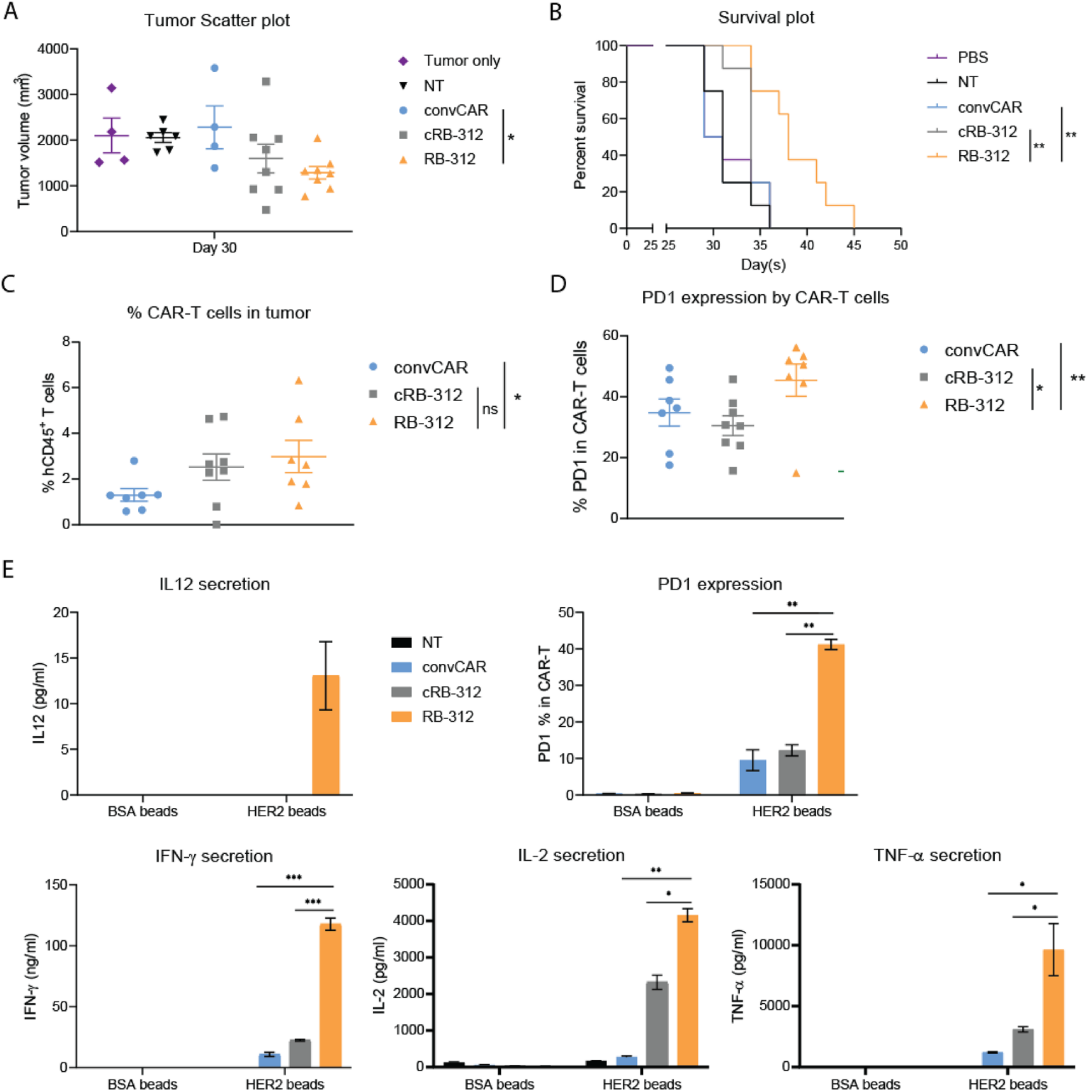
Intratumoral effectiveness of RB‐312 against FaDu xenograft. **A** Scatter plot for tumor growth at day 30 (0.3 M CAR‐T cell dose). **B** RB‐312 effect on survival—survival analysis until day 45. Survival was significantly increase in mice injected with RB-312. **C** Intratumoral persistence of CAR‐T cells at necropsy **D** PD1 expression by intratumoral CAR-T cells at necropsy showed that RB-312 displays significantly higher PD1 surface markers. **E** Retention samples from the animal study were stimulated with HER2-coated beads and analysis of IL-12/p70 expression, PD1 expression, IFN‐γ, IL-2 and TNF‐α secretion was performed

Next, RB-312, cRB-312 and convHER2 CAR (convCAR) T cells, were administered in vivo in NOD-SCID-IL2rγ−/− (NSG) mice subcutaneously implanted with FaDu cells. To test efficacy independent of systemic variables affecting trafficking and homing, CAR-T cells were intratumorally injected and tumor growth was monitored (Fig. 4). RB-312 demonstrated stronger reduction of tumor growth and better colonization of tumors by residual CAR-T cells compared to convCAR T cells. No significant differences were observed in tumor colonization between RB-312 and cRB-312 (Fig. 4A and C). This is likely due to the overexpression of LAT peculiar to the LdCV construct, known to enhance independently T cell activation and persistence [27]. However, significantly prolonged survival was observed in mice receiving RB-312 compared to both convCAR and cRB-312 (Fig. 4B).

Interestingly, RB-312 expressed significantly higher levels of PD-1 at necropsy compared to convCAR and cRB-312 (Fig. 4D) suggesting that IL-12 may expedite CAR-T cells differentiation and exhaustion as a consequence of stronger activation. Higher PD-1 expression was also observed in tumor-infiltrating CAR-T cells in mice implanted with FaDu_IL12 tumor xenograft compared to that of FaDu tumor implant, confirming the effect was due to IL-12 presence rather than signaling alteration peculiar to the LdCV system. To further confirm these observations, CAR-T cells retained for testing at the time of infusion (*retention samples*) from the RB-312 animal study were stimulated with HER2-coated beads. Significantly higher PD-1 expression was observed in RB-312 compared to respective controls. RB-312 again showed significantly higher production of homeostatic cytokines (Fig. 4E). These data support the notion that IL-12 is a potent inducer of PD-1 expression in T cells because of stronger T cell activation. This limits at the same time their anti-tumor efficacy by rendering them vulnerable to PD-L1 suppression.

### Low-dose IL-12/p70 by RB-312 synergize with anti-PD-L1 to induce superior tumor regression

RB-312 demonstrated potent activation of effector functions but as a drawback vulnerability to checkpoint engagement, in particular through the interaction between PD-1 and PD-L1 leading to a prematurely exhausted phenotype. This is especially the case in tumors with high density of PD-L1 expression as pressure tested in this animal model. To stress this potential inhibitory mechanism, FaDu tumor cells were engineered to overexpress PD-L1. The potential benefit of a combination with checkpoint inhibition (to block the PD-1/PD-L1 interactions) was then investigated.

**Fig. 5:**
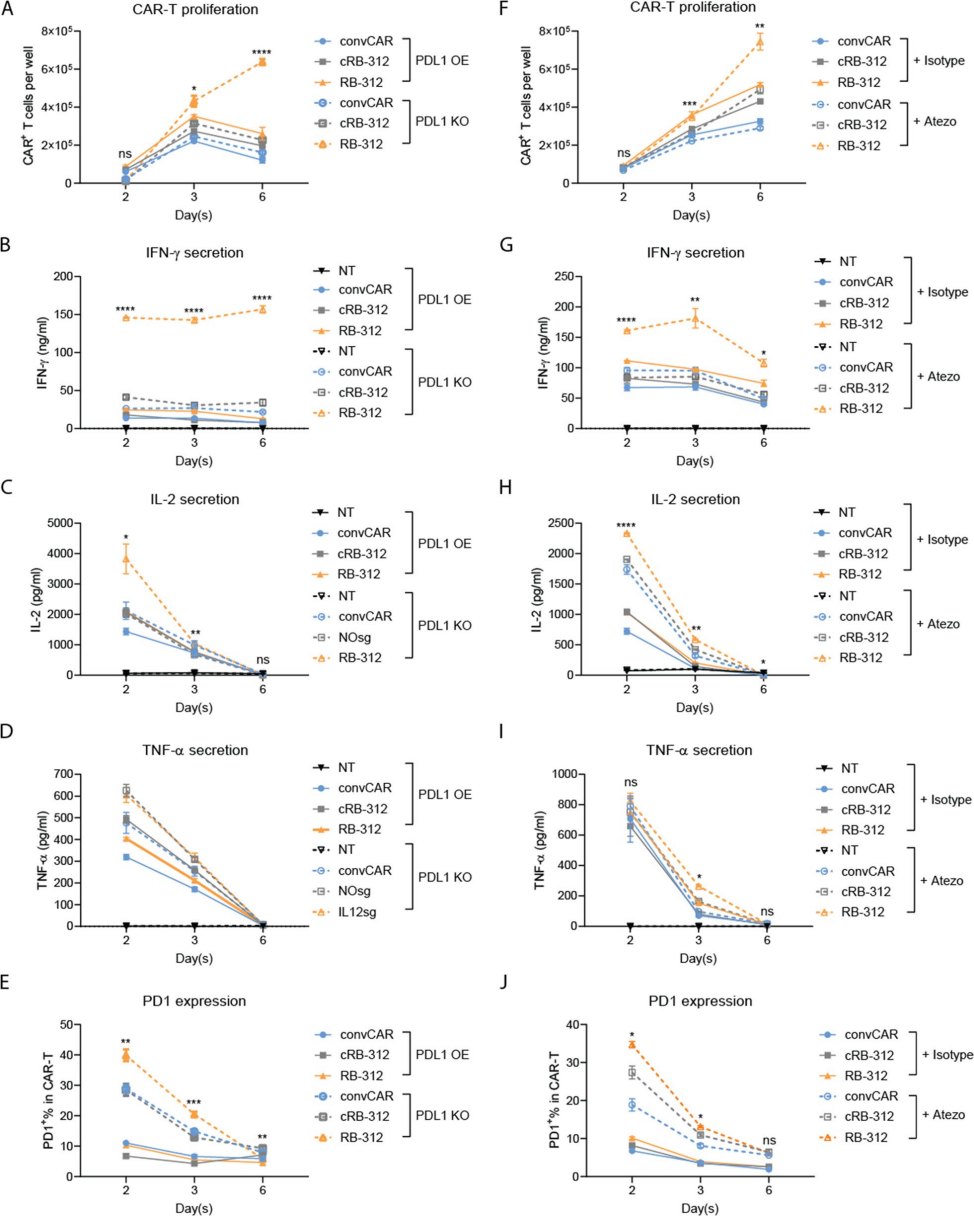
Kinetics of RB-312 cellular function in combination with anti-PD-L1. NT, convCAR, cRB-312 and RB‐312 were stimulated with either FaDu cells overexpressing PDL1 (PDL1 OE) or FaDu engineered with PDL1 knockout (PDL1 KO; **A**–**E**), or stimulated with FaDu cells with and without addition of atezolizumab (10 ug/ml, **F**–**J**) at 3:1 E:T ratio for 6 days. Representative kinetics of CAR-T proliferation, IFN‐γ, IL-2, TNF‐α secretion and PD1 expression were obtained at day 2, 3 and 6 as indicated

To test the dominance of this immune suppressive mechanism, we first generated a FaDu tumor line with PD-L1 knocked out by CRISPR. We then compared the expansion and cytokine profiles of RB-312 upon co-cultured with FaDu carrying PD-L1 overexpression (PDL1/OE) or PD-L1 knockout (PDL1/KO) at E:T ratio 3:1. As shown in Fig. 5A and B, RB-312 showed significantly better CAR-T expansion and IFN-γ secretion upon co-culture with PDL1/KO (orange dashed line) compared to convCAR and cRB-312 (blue and grey dashed lines) and co-culture with PDL1/OE (orange solid line). IL-2 secretion was also higher in RB-312 when co-culture with PDL1/KO compared to respective controls, although no significant differences were seen between RB-312 and respective controls upon stimulation by PDL1/OE (Fig. 5C). TNF-α secretion in RB-312 was higher than convCAR T cells but not cRB-312 co-cultured with PDL1/OE or PDL1/KO (Fig. 5D).

To test the synergy of RB-312 activation combined to PD-L1 blockade, we set up the same co-culture assay using the PD-L1 blocking antibody—atezolizumab (***atezo***, dashed lines) compared to an isotype antibody (solid lines). Again, PD-L1 blockade by atezo enhanced CAR-T expansion, IFN-γ and IL-2 secretion in RB-312 but not TNF-α when compared to atezo-treated convCAR T cells or cRB-312 (Fig. 5F–I). Moreover, PD-L1 blockade by knock-out or atezo treatment showed the same trend of significantly enhanced PD-1 expression in RB-312 (Fig. 5E and J) indicating increased T cell activation upon PD-L1 blockade.

**Fig. 6:**
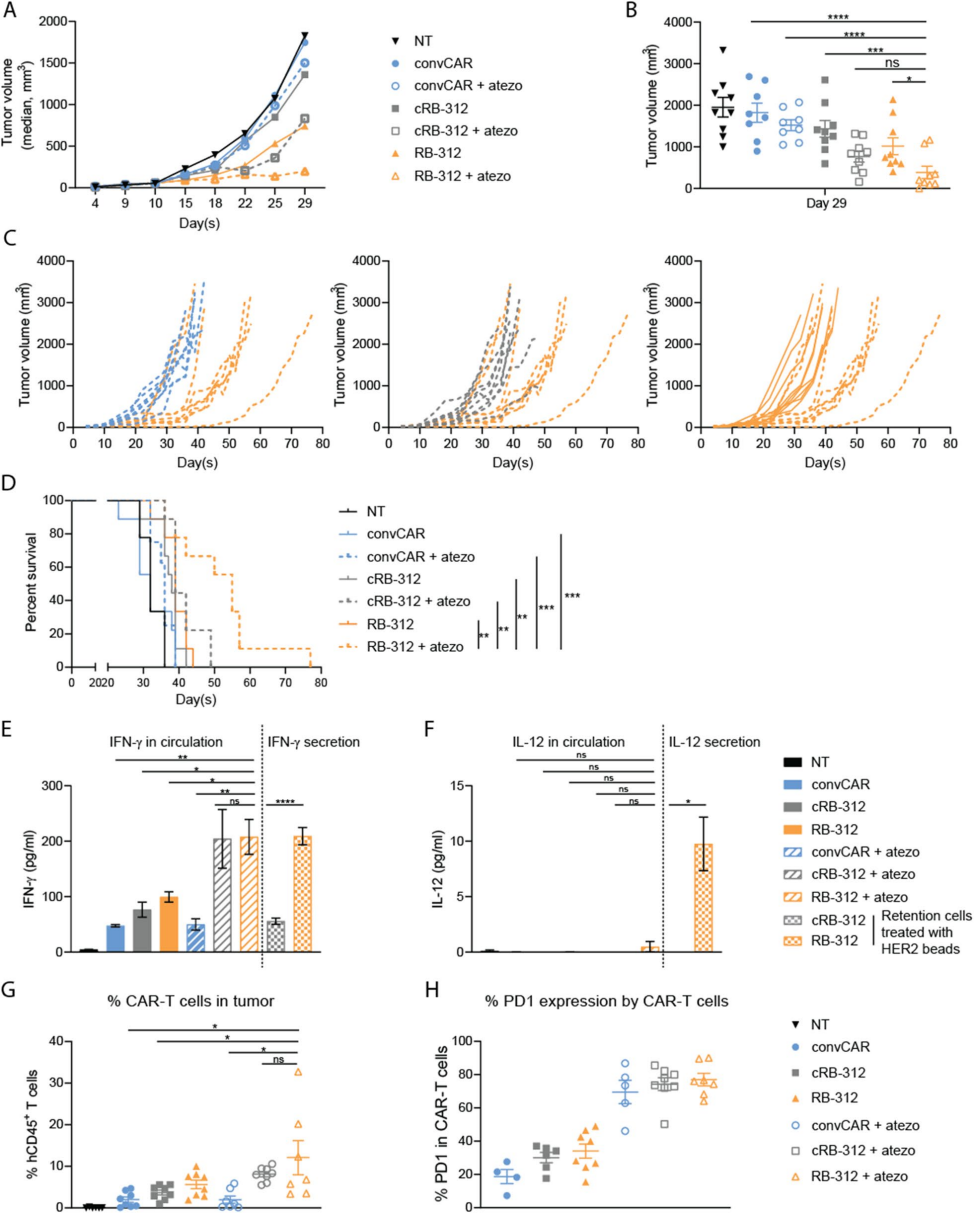
Synergistic effectiveness of RB-312 with anti-PD-L1 against FaDu xenograft. **A** Mice intratumorally dosed with RB‐312 or respective control groups were administrated with or without atezolizumab and average tumor growth was followed till day 29. **B** After day 29, mice in control groups began to die and average growth is not informative. Scatter plots were subsequently used as exemplified for day 29. **C** RB‐312 effect on survival of individual mice and **D** cumulative survival—survival analysis until day 77. **E** Quantification of IFN‐γ systemic levels (left) and quantification of IFN‐γ secretion (right) after HER2 beads stimulation of retention samples cRB-312 and RB-312. **F** Quantification of IL-12 systemic levels (left) and quantification of IL-12 secretion (right) after HER2 beads stimulation of retention samples cRB-312 and RB-312. **G** Intratumoral persistence of hCD45^+^ CAR‐T cells and **H** percentage of PD‐1 expressing hCD45^+^ CAR‐T cells at necropsy

We then explored the impact of PD-L1 blockade as a strategy to enhance the efficacy of RB-312 in vivo (Additional file 1: Fig. S2). A significant reduction in tumor progression after combinatorial treatment with intra-tumoral administration of RB-312 and systemic delivery of atezo was observed (Fig. 6A–C). Thirty days following adoptive transfer of CAR-T cells, numerous mice from the control groups began to die while survival was significantly prolonged in the RB-312 plus atezo group (orange dashed line) compared with the other groups (Fig. 6D). Such a synergistic effect was associated with elevated level of circulating IFN-γ (Fig. 6E). Importantly, circulating IL-12 was undetectable suggesting safety of nanoscale delivery of IL-12 by RB-312 (Fig. 6F).

At necropsy, the RB-312 with atezo combination was associated with a significant increase in the frequencies of colonized tumor infiltrating CAR-T cells, compared to groups with isotype addition or convCAR with atezo, but not cRB-312 with atezo addition (Fig. 6G). Again, groups with PD-L1 blockade were characterized by increased expression of PD-1 (Fig. 6H). In summary, these findings suggest that low-dose IL-12 by RB-312 synergizes with anti-PD-L1 to induce superior tumor suppression when secreting nanoscale levels of IL-12 undetectable in the peripheral circulation.

## Discussion

Effective cell therapy of cancer should challenge the disease simultaneously through multiple modalities to address cell fitness [4, 28, 29], and resilience in a metabolically and immunologically hostile TME [3, 30, 31]. Recent advances in synthetic biology allow improvements of CAR architecture to prolong CAR-T cell persistence in vivo and enhance efficacy as previously shown by reversible prevention of PD-1 expression with CRISPRi in response to antigen encounter through [13].

Here, we exploited a similar design replacing CRISPRi with CRISPRa to create RB-312, a novel CAR-T product that allows antigen-dependent release of nanoscale level of endogenous IL-12. RB-312 is activated exclusively upon antigen encounter and, once the antigenic stimulus is eradicated, it is switched off thus limiting to the minimum the risk of systemic toxicity typically observed when this potent cytokine is employed in clinical settings [16, 23]. In fact, no circulating IL-12 levels could be detected in vivo when a therapeutically effective dose of RB-312 was tested in combination with checkpoint blockade (Fig. 6F).

This platform targets the regulatory region of endogenous genes through epigenetic modulation without any transient or permanent architectural changes of genomic DNA. This contrasts with the traditional genome-editing tools, including CRISPR, TALENS and ZFNs, which involves double strand break and irreversible chromosomal editing [9]. Moreover, the CRISPR activation platform allows simultaneous targeting of several endogenous genes with multiple sgRNAs cloned in the same vector. Indeed, preliminary results (not shown) demonstrated that up to three sgRNAs can be multiplexed in the same vector, leading to successful upregulation of three proteins.

This proof-of-concept study suggests that the CRISPR activation platform is a valid and safe alternative to the engineered, armored CAR-T cells. We first demonstrated that, with this platform, it is possible to fine tune the upregulation of IL-12 using different RNA polymerase III promoters to drive sgRNA expression, leading to high or low IL-12 production. Through the optimization of the sgRNA scaffold region, we were able to achieve further enhanced IL-12 expression (data not shown). Furthermore, our data suggests that through selection of the sgRNAs targeting at different sites the TSS, it is possible to induce the expression of various quantities of IL12A and IL12B.

Due to the well-known systemic toxicity observed in patients receiving IL-12 [8, 23, 32], we focused on a “least minimum denominator” approach to ensure that RB-312 delivers nanoscale levels of IL-12/p70 only within the TME just sufficient to produce the anti-cancer effects. We showed that RB-312 conditionally activates IL-12 upon exposure to HER2 expressing cancer cells, resulting in better CAR-T cell expansion in vitro and improved antitumor activity in vivo. Our data indicate that IL-12 can shape the TME at even low-dose and reprogram it towards an environment conducive to T cell-mediated anti-tumor immunity. Indeed, the limited efficacy observed was due to the natural induction of PD-1 expression caused by this cytokine. This could in turn be overcome by checkpoint blockade with atezolizumab. We recognize that this model may overstated the role of PDL-1 expression and further studies will be necessary to elucidate and fine-tune the pharmacodynamics of this interactions in conditions closer to the natural behavior of cancer cells exposed to IFN-γ produced by CAR-T cells.

Several studies, however, support this combination showing that, while treatment with IL-12 alone is not sufficient to eradicate tumors, combination with checkpoint inhibitors results in eradication of cancer in preclinical models [33–37] and in early phase clinical trials [38–40].

In summary, we provided the first line of evidence supporting the use of synthetic CRISPR activation as an efficient tool for T cell engineering that allows the conditional and regulated expression of endogenous genes. We created a synthetic CAR-T cell product, RB-312, as the first application of CRISPR activation in the prospect of a future clinical study.

## Material and methods

### Lentiviral vector construction and production

The lentiviral vector was designed as previously described [13, 41]. LdCV vector contains the following: human codon-optimized *S. pyogenes* dCas9 which was fused at the C-terminus with VP64-p65-Rta (VPR). LAT-TCS-dCas9-VPR was assembled by fusing LAT (Human cDNA, NM_001014987.2) with dCas9-VPR-Q8 and cloned into a modified pHR-SFFV lentiviral vector (PMID: 26830878). SFFV promoter was replaced with EF1α promoter. TCS sequence (ENLYFQ) was inserted in between LAT and dCas9 and was flanked by GS linker. Two nuclear export signals (NES, LALKLAGLDI and LQLPPLERLTL) flanked LAT to ensure the cytoplasmic localization of the chimera protein.

The second vector harbors the HER2-specific CAR linked to the tobacco etch virus (TEV) protease (PMID: 29263378), which was designed as previously described (PMID: 34743703). For HER2 CAR detection and enrichment, P2A-tNGFR (truncated NGFR) was fused C-terminal to TEV. IL-12A sgRNA (IL12Asg) and IL-12B sgRNA (IL12Bsg) were controlled by human U6 promoter (hU6) and H1 promoter, respectively. Guide RNAs were cloned into HER2 CAR-TEV vector upstream of the EF1α promoter for the ease of engineering the ChaCha system into human primary T cells (PMID: 29263378). For multiplexing experiments, IL12Asg and IL12Bsg in tandem with described promoters, were cloned into pHREF1αp lentiviral vector expressing HER2 CAR-TEV.

Second-generation, self-inactivating lentiviral supernatant was produced in the HEK293 T packaging cell line as previously described (PMID: 34743703). The 72-h viral supernatants were harvested, filtered through 0.45 μm PVDF membrane filter unit, layered on top of 10% sucrose solution, and followed by high-speed centrifugation at 10,000*g* for 16 h at 4 °C. The concentrated lentiviral pellet was resuspended on RPMI media and frozen at − 80 °C for future use.

Lentiviral titration was performed in 96 well flat bottom treated plate. HT1080 cells were adjusted at a density of 500,000 cells/ml and 4 ml of cell mix were combined with 16 μl of Transplus™ (Alstem). The mix was aliquoted in 100 μl/well and incubated at 37 °C for one hour. Crude and concentrated virus were thawed on ice. Four serial dilutions were performed, mixing 15 μl of virus and 135 μl of cell media. 100 μl of each dilution were then transferred onto seeded cells and incubated 3 days at 37 °C. Cells were then washed with PBS, trypsinized and incubated with 100 μl of antibody mixture NGFR-FITC (clone ME20.4, Biolegend 1:100) and Q8-PE (clone QBEND/10, ThermoFisher 4:100) for 30 min at 4 °C. Cells were washed twice in FACS buffer and resuspended in 200 μl FACS buffer. Samples were run in cytoFlex (Beckman) and FITC or PE positive cells were screened. For titer calculation selected wells (for dilution factor) presented 1–20% fluorescent positive cells and titer was calculated using the following formula:

$$\mathrm{Titer }\,(\mathrm{TU}/\mathrm{ml}) = (50.000 \times \mathrm{ \%positive}) / (100 \times \mathrm{ fold\, dilution}).$$

### IL-12 sgRNA design and screening

An arrayed screen for IL-12 sgRNAs was performed. IL-12A and IL-12B sgRNA libraries were designed as previously described [13]. The screening of IL-12 was performed by transfection of 26 sgRNA for p35 and 31 sgRNA for p40 into dCas9-VPR expressing Jurkat cells. The top 5 candidates for IL-12A and IL-12B (highest knockdown and fewest computationally predicted off-targets) were further tested in primary T cells. The selected top candidates were cloned into pHR-EF1αp lentiviral vector expressing HER2 CAR-TEV as described above.

### Primary T cell isolation and CAR T cell production

Primary T cell isolation and CAR-T cells production were performed as previously described [13]. At day 1 after activation, T cells were transduced with lentiviral vectors carrying LAT-dCas9VPR-Q8 (at MOI = 10), followed by a second transduction with HER2 CAR-TEV-tNGFR/IL-12sgRNAs (at MOI = 10) at day 2. At day 5, T cells activation was removed, and transduced T cells were cultured in a 24-well plate. At day 6, double-transduced cells were enriched for double positive, through cell sorting (Sony Cell Sorter) for Q8 and tNGFR. After cell sorting, T cells were cultured up to day 14 in T cell culture media with IL-15 and IL-7 (10 ng/ml). CAR-T cells were used for in vitro assays or implanted into mice at day 14 of manufacturing run.

### Cell lines

Human embryonic kidney 293 cells (HEK293T), human fibrosarcoma (HT1080) and cells from hypopharyngeal tumor of a squamous cell carcinoma patient (FaDu) were obtained from ATCC (Manassas, VA). FaDu-PD-L1 (named FaDu-PDL1) was generated as previously reported [13].

All cells were routinely tested using the MycoAlert Mycoplasma Detection Kit (Lonza).

### Quantification of HER2.CAR and dCas9 vector copies in transduced T cells

Genomic DNA was extracted from engineered cells using DNeasy blood and tissue kit (Qiagen). PCR amplification was performed using primers for RRE sequence in the lentiviral backbone, for CAR and LdCV. RPP30 gene was used as reference control. Droplets were generated using the QX200 droplet generator (Bio-Rad) according to the manufacturer protocol. Amplification in droplets was measured using QX200 Droplet reader (Bio-Rad) and analyzed using Quantasoft software.

### Co-culture experiments and cytokine production

Supernatant from co-cultured cells was harvested and analyzed for human IFN-γ, IL-2 and TNF-α by ELISA (Biolegend) and human IL-12 by Quantikine HS ELISA (R&D system).

### HER2 micro‑bead preparation and HER2 beads stimulation

Beads were prepared as previously reported [13, 26].

Approximately 80,000 CAR-T cells were cultured in 96-well flat bottom plates and HER2 beads were added at a 1:1 bead to HER2 CAR-T cells ratio, in TBM media. Three days after beads stimulation, CAR-T cells were harvested and analyzed.

### Animal experiments

After counting, 0.5 × 10^6^ FaDu tumor cells were re-suspended in 100 µl of PBS plus matrigel (Corning) and subcutaneously injected into the right flank of 6-to-8-week-old female immune deficient NSG mice (JAX laboratory). When average tumor size reached close to 100 mm^3^, mice were randomized into different groups (see Additional file 1: Fig. S2 for details), and relevant mice received anti-PD-L1 (atezolizumab, 10 mg/kg) intravenously. At the following day, a total of 0.3 × 10^6^ of HER2 CAR-T cells, as specified in the figure legends, were injected intratumorally in a volume of 20 µl. Mice in the atezolizumab group were continuously treated with atezolizumab (5 mg/kg) twice every week. Tumor dimensions were measured biweekly with digital calipers, and tumor volumes were calculated using the formula *V* = ½ (length × width^2^). Mice were humanely euthanized according to IACUC protocol and tumor were resected immediately after euthanasia for further analysis.

### Isolation of tumor‑infiltrating CAR‑T cells

Solid tumor tissue was collected, rinsed with PBS, and mechanically dissociated using the Miltenyi gentleMACS dissociator. The cell suspension was filtered using a MACS SmartStrainer, the strainer was washed with 10 ml of RPMI media and samples were centrifuged 500*g* for 5 min. Pellet were resuspended in 1.5 ml of CS10 freezing media and 300 ul were dispensed in each cryovial. Single-cell suspensions were subsequently thawed and stained using the described antibodies and analyzed by flow cytometry.

### Flow cytometry

Human HER2 and PD-L1 expression on tumor cells was detected using human HER2-PE-CY7 (clone 24D2, Biolegend) and PD-L1-APC (clone MIH1, eBioscience). Human T cells surface phenotype and transduction efficiency were assessed using the following antibodies: NGFR-FITC (clone ME20.4, Biolegend), Q8 (clone QBEND/10, ThermoFisher), CD45-AF700 or CD45-BV605 (clone HI30, Biolegend), CD3-APC (clone SK7, Biolegend), CD4-PerCP (clone SK3, Biolegend), CD4-BB700 (clone SK3, BD Bioscience), CD8-BV510 (clone SK1, Biolegend), CD27-PE-CY7 or BV786 (clone M-T271, Biolegend), CD28-BV605 or BV711 (clone CD28.2, Biolegend). Expression of T cell inhibitory receptors was analyzed using PD-1-BV421 or PD-1-BV605 (clone EH12.2H7, Biolegend), TIM-3-BV605 or TIM-3-PE-CY-7 (clone F38-2E2, Biolegend), CD39 (clone A1, Biolegend), LAG-3-BV711 (clone 11C3C65, Biolegend). Live/dead discrimination was determined using LIVE/DEAD fixable Near-IR dead cell stain kit (ThermoFisher). Mouse cells were assessed using the following antibodies: Flow cytometry results were analyzed using Kaluza software (Beckman Coulter).

### Statistical analysis

Unpaired two-tailed t-test was used to analyze significant differences between groups. Statistical analyses were performed using GraphPad Prism8. Survival curve were compared using the log-rank Mantel-Cox test.

Significance of findings was defined as: ns not significant; *p ≤ 0.05; **p ≤ 0.01; ***p ≤ 0.001, ****p ≤ 0.0001.

## Supplementary Information

**Additional file 1: Fig. S1 A.** IL-12/p70 secretion quantification of FaDu cell line overexpressing both PD-L1 and IL-12 (FaDu_IL12). **B-F,** Cytotoxicity activity, CAR-T proliferation and IFN‐γ, IL-2, TNF‐α secretion in NT and convCAR stimulated with FaDu or FaDu_IL12 at 1:5 effector-to-target ratio for 2, 3 and 6 days.** G,** Percent of survival of NSG mice subcutaneously implanted with FaDu and FaDU_IL12 cells and treated with convCAR and NT (0.3 M cells). **H,** Surface marker PD-1 quantification in convCAR cocultured with FaDu or FaDu_IL12 tumor cells. **Figure S2 A,** Intratumoral administration model and study design **B and C,** Experimental set up—All treatment groups included eight mice (in B, for Fig. 4) or nine mice (in C, for Fig. 6) receiving subcutaneous implantation of 0.5 million FaDu cells followed by adoptive transfer of CAR-T cells at day 10. Atezolizumab (10 mg/kg) was administered intravenously in the relevant groups the day before adoptive transfer (day 9) and subsequently at 5 mg/kg twice a week.

## Data Availability

All data relevant to the study are included in the article or uploaded as supplementary information. Data are available on reasonable requests.

## Abbreviations

Atezo: Atezolizumab
BSA: Bovine serum albumin
CAR: Chimeric antigen receptor
convCAR: Conventional CAR
cRB-312: Control CAR construct analog to RB-312 but without sgRNAs
CRISPR: Clustered regularly interspaced short palindromic repeats
CRISPRa/i: CRISPR activation or interference
dCAS: Nuclease deactivated CRISPR-associated system
FaDu: PDL-1 overexpressing hypopharyngeal carcinoma cell line
FaDu_IL-12: Constitutively IL-12-expressing FaDu cell line
HER2: Human epidermal growth factor
HER2-TEV: Lentiviral construct containing the HER2-CAR and protease containing construct
IFN: Interferon
IL: Interleukin
ITAM: Immunoreceptor tyrosine-based activation motif
KRAB: Kruppel associated box domain
LAT: Linker for activation of t cells
LdCV: Lentiviral construct containing LAT-dCAS, and VPR
MDA-MB-231: Metastatic triple negative breast cancer cell line
NSG: NOD-SCID-IL2rγ−/− mice
PD1: Programmed cell death 1
PDL1: Programmed death ligand 1
PDL1/KO: PDL1 knock out cell line
PDL1/OE: PDL1 over expression cell line
qPCR: Real-time quantitative polymerase chain reaction
RB-312: HER2 CAR armed with conditional secretion of endogenous IL-12
scFv: Single chain variable fragment
sgRNA: Single guide RNA
TCS: TEV-cleavable sequence
TEV: Tobacco etch virus
TBM: T cell basal media
TME: Tumor microenvironment
TNF: Tumor necrosis factor
TSS: Transcription start site
VPR: VP64-p65-Rta promoter
ZFN: Zinc finger nuclease
