# Supplementary material for: Nanoscale, antigen encounter-dependent, IL-12 delivery by CAR T cells plus PD-L1 blockade for cancer treatment
Source: J Transl Med. 2023 Feb 28;21:158. doi: 10.1186/s12967-023-04014-9 (PMC9976446; doi:10.1186/s12967-023-04014-9)
Supplement: Supplementary file 1 — Additional file 1: Fig. S1 A. IL-12/p70 secretion quantification of FaDu cell line overexpressing both PD-L1 and IL-12 (FaDu_IL12). B-F, Cytotoxicity activity, CAR-T proliferation and IFN‐γ, IL-2, TNF‐α secretion in NT and convCAR stimulated with FaDu or FaDu_IL12 at 1:5 effector-to-target ratio for 2, 3 and 6 days. G, Percent of survival of NSG mice subcutaneously implanted with FaDu and FaDU_IL12 cells and treated with convCAR and NT (0.3 M cells). H, Surface marker PD-1 quantification in convCAR cocultured with FaDu or FaDu_IL12 tumor cells. Figure S2 A, Intratumoral administration model and study design B and C, Experimental set up—All treatment groups included eight mice (in B, for Fig. 4) or nine mice (in C, for Fig. 6) receiving subcutaneous implantation of 0.5 million FaDu cells followed by adoptive transfer of CAR-T cells at day 10. Atezolizumab (10 mg/kg) was administered intravenously in the relevant groups the day before adoptive transfer (day 9) and subsequently at 5 mg/kg twice a week. [file 12967_2023_4014_MOESM1_ESM.docx]

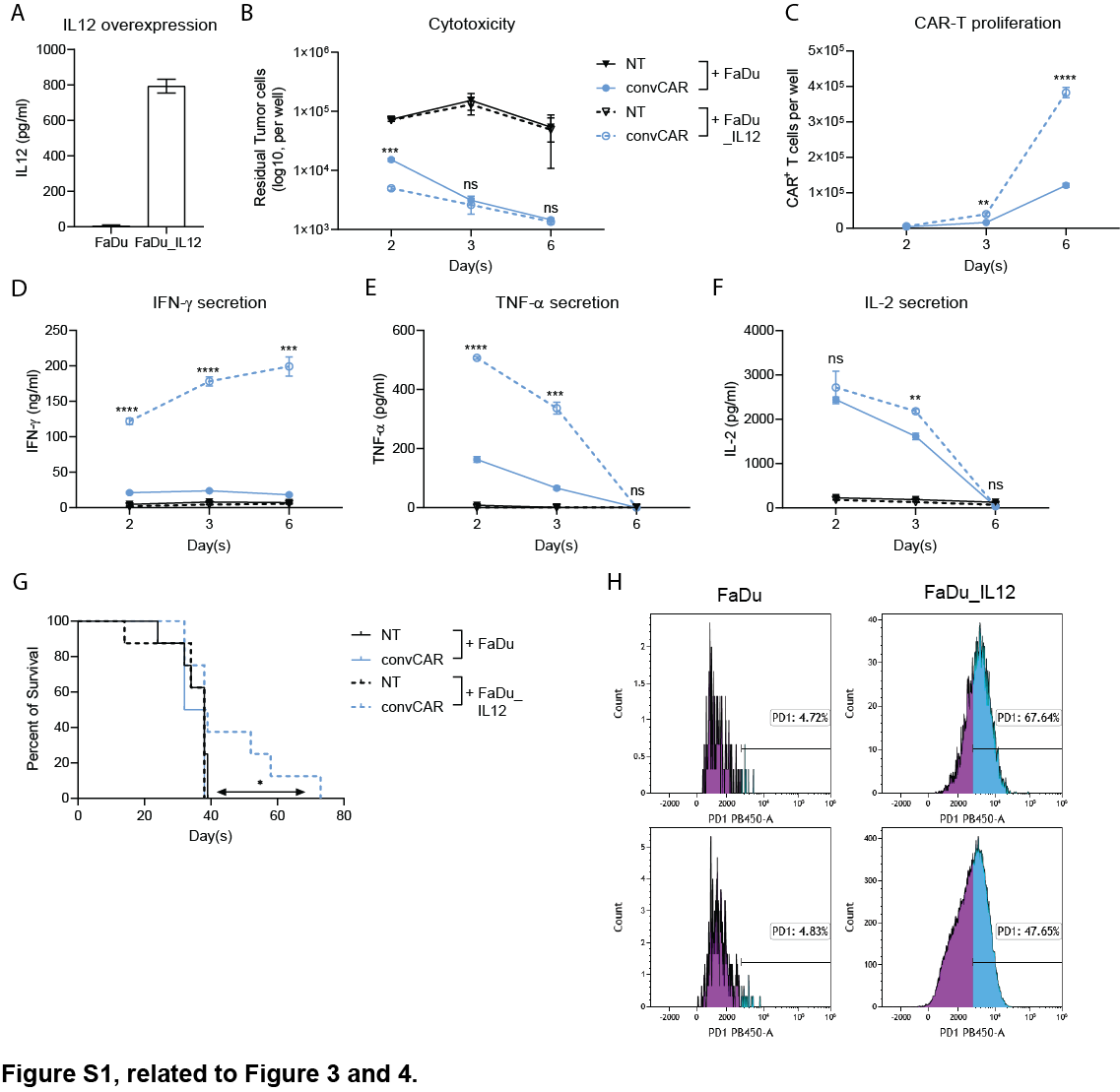


**Fig. S1 A,** IL-12/p70 secretion quantification of FaDu cell line overexpressing both PD-L1 and IL-12 (FaDu_IL12). **B-F,** Cytotoxicity activity, CAR-T proliferation and IFN‐γ, IL-2, TNF‐α secretion in NT and convCAR stimulated with FaDu or FaDu_IL12 at 1:5 effector-to-target ratio for 2, 3 and 6 days. **G,** Percent of survival of NSG mice subcutaneously implanted with FaDu and FaDU_IL12 cells and treated with convCAR and NT (0.3 M cells). **H,** Surface marker PD-1 quantification in convCAR cocultured with FaDu or FaDu_IL12 tumor cells.

**Figure S2 A,** Intratumoral administration model and study design **B and C,** Experimental set up – All treatment groups included eight mice (in B, for Figure 4) or nine mice (in C, for Figure 6) receiving subcutaneous implantation of 0.5 million FaDu cells followed by adoptive transfer of CAR-T cells at day 10. Atezolizumab (10 mg/kg) was administered intravenously in the relevant groups the day before adoptive transfer (day 9) and subsequently at 5 mg/kg twice a week.
